# Development of new physical activity and sedentary behavior change self-efficacy questionnaires using item response modeling

**DOI:** 10.1186/1479-5868-6-20

**Published:** 2009-03-31

**Authors:** Russell Jago, Tom Baranowski, Kathy Watson, Christine Bachman, Janice C Baranowski, Debbe Thompson, Arthur E Hernández, Elizabeth Venditti, Tara Blackshear, Esther Moe

**Affiliations:** 1 Department of Exercise, Nutrition & Health Sciences, University of Bristol, Tyndall Avenue, Bristol, BS8 1TP, UK; 2 USDA/ARS Children's Nutrition Research Center, Department of Pediatrics, Baylor College of Medicine, 1100 Bates Street, Houston, TX 77030-2600, USA; 3 Department of Psychology, University of Houston-Downtown, Houston, TX, USA; 4 College of Education, Texas A&M University, Corpus Christi, College Station, USA; 5 University of Pittsburgh, Pittsburgh, PA, USA; 6 School of Nursing, University of North Carolina at Chapel Hill, Chapel Hill, USA; 7 Division of Health Promotion and Sports Medicine, Oregon Health & Sciences University, Portland, OR, USA

## Abstract

**Background:**

Theoretically, increased levels of physical activity self-efficacy (PASE) should lead to increased physical activity, but few studies have reported this effect among youth. This failure may be at least partially attributable to measurement limitations. In this study, Item Response Modeling (IRM) was used to develop new physical activity and sedentary behavior change self-efficacy scales. The validity of the new scales was compared with accelerometer assessments of physical activity and sedentary behavior.

**Methods:**

New PASE and sedentary behavior change (TV viewing, computer video game use, and telephone use) self-efficacy items were developed. The scales were completed by 714, 6^th ^grade students in seven US cities. A limited number of participants (83) also wore an accelerometer for five days and provided at least 3 full days of complete data. The new scales were analyzed using Classical Test Theory (CTT) and IRM; a reduced set of items was produced with IRM and correlated with accelerometer counts per minute and minutes of sedentary, light and moderate to vigorous activity per day after school.

**Results:**

The PASE items discriminated between high and low levels of PASE. Full and reduced scales were weakly correlated (r = 0.18) with accelerometer counts per minute after school for boys, with comparable associations for girls. Weaker correlations were observed between PASE and minutes of moderate to vigorous activity (r = 0.09 – 0.11). The uni-dimensionality of the sedentary scales was established by both exploratory factor analysis and the fit of items to the underlying variable and reliability was assessed across the length of the underlying variable with some limitations. The reduced sedentary behavior scales had poor reliability. The full scales were moderately correlated with light intensity physical activity after school (r = 0.17 to 0.33) and sedentary behavior (r = -0.29 to -0.12) among the boys, but not for girls.

**Conclusion:**

New physical activity and sedentary behavior change self-efficacy scales have fewer items than classical test theory derived alternatives and have reasonable validity for boys, but more work is needed to develop comparable scales for girls. Fitting the items to a underlying variable could be useful in tailoring interventions to this scale.

## Background

Regular physical activity is associated with a decreased risk of a number of diseases including type 2 diabetes [1], cardiovascular disease [2], and some cancers [3,4]. Numerous studies have shown that most children and adolescents in the UK and US do not engage in sufficient amounts of physical activity to meet their national physical activity guidelines [5-7]. The mediating variable model [8,9] suggests that change in youth physical activity is most likely to be achieved by identifying and targeting mediators shown to influence physical activity. Applying psychological theories to intervention design allows researchers to identify and manipulate key mediators systematically as a means of changing behavior [9].

Social cognitive theory [10,11] has been used extensively to understand youth physical activity. Self-efficacy which can be interpreted as a person's perceived competence to engage in an activity, is a central component of social cognitive theory [11] and numerous studies have reported positive correlations between physical activity self-efficacy (PASE) and physical activity among youth [12-14]. Despite these associations, few studies have reported that self-efficacy functioned as a mediator of physical activity change among either youth [15] or adults [16,17]. The absence of a self-efficacy mediating effect may be caused by either an inability of the intervention to change self-efficacy or failure of the assessment methods to accurately capture change in self-efficacy.

Current physical activity self-efficacy (PASE) scales have been developed and tested using classical test theory (CTT) [18,19]. CTT sub-scales are usually identified through principal component analyses; test re-test reliability is obtained through correlational analyses; and the internal consistency (reliability) of the scales and sub-scales is obtained using Cronbach's alpha [20]. Item Response Modeling improves on these methods by linking the individuals' difficulty of response to each item [20]. Item difficulty is particularly important as it allows the researcher to ensure that the items included in a scale measure a range of difficulty options (from easy to hard) reflecting the distribution of respondents across the scale. Items with similar levels of difficulty are comparable, meaning items at one point on the scale are overlapping. Identifying overlapping items makes it possible to produce a questionnaire with fewer items, thus reducing participant burden. The aims of this paper were to: 1) use IRM to assess new physical activity and sedentary behavior change (TV viewing, computer video game use, and telephone use) self-efficacy scales; and 2) assess the validity of the new scales in comparison with assessments of physical activity and sedentary behaviors; and 3) reduce the items and test the resulting scales.

## Methods

### Participants

The data presented were collected as part of preliminary work performed for the Studies to Treat or Prevent Pediatric Type 2 Diabetes – Prevention (STOPP-T2D). STOPP-T2D – Prevention is a multi-site study funded by the National Institute of Diabetes and Digestive and Kidney Diseases that is designed to reduce the risk factors for type 2 diabetes among middle school children. Participants were 6^th ^grade students recruited from seven middle schools. Each school was recruited by one of the seven field centers in Houston (Baylor College of Medicine, Houston TX), California (University of California Irvine, Irvine CA), North Carolina (University of North Carolina at Chapel Hill, Chapel Hill NC), Oregon (Oregon Health & Sciences University, Portland OR), Pittsburgh (University of Pittsburgh, Pittsburgh PA), Philadelphia (Pennsylvania University, PA) and San Antonio (University of Texas Health Science Center – San Antonio TX). The study was coordinated by the Biostatistics Center at George Washington University, Rockville MD. Schools were required to have at least 40% of the participants enrolled in the school to be from an ethnic group known to be at increased risk of type 2 diabetes mellitus (African American, American Indian or Hispanic) [21]. Ethical approval was obtained from the Institutional Review Board of each field center and the Coordinating Center, and written informed consent and assent were obtained for all participants prior to participation.

### Scale development & data collection

In earlier work with a children's diet self-efficacy scale, we found that the scale only measured a narrow range on the difficulty dimension in comparison to participants, many of whom had higher or lower levels of self-efficacy [22]. Items should cover the full range of difficulty to ensure content validity. To avoid this problem for the current PASE scale, we started with previous physical activity and sedentary behavior self efficacy scales [18]. Following theory specified procedures [23], easy and difficult versions for each behavior were then generated for each item. For example, engaging in physical activity for 30 minutes after school on either one day (easy) or on four days (hard). This process was started by one of the co-authors (CB) and then reviewed by several of the other authors (RJ, TB, JB, DT) as a multidisciplinary expert panel. Several iterations of item development were conducted until all were satisfied with the items. The questionnaire included 25 physical activity items and 8 items for each of the three sedentary behaviors. Each item asked "How sure are you that you can ....", with sure and not sure as response categories. The specific items appear in Table 1 and Table 2. The dichotomous (sure and not sure) response categories were selected based on our previous work in this age group that empirically demonstrated that self-efficacy Likert responses can be reduced to dichotomous outcomes without a loss of information [22], thereby keeping respondent options simple. Next, cognitive interviewing was conducted with these items to be sure that the target aged children understood the items and response scale as intended. This provided additional revisions and multidisciplinary reviews. Items were loaded onto Palm Pilots that were then completed by participants at the schools and then downloaded into a central database. The 23 physical activity self-efficacy items and 24 physical inactivity items were collected as part of a larger data collection effort to develop psychosocial questionnaires using item response modeling, with participants asked to complete a total of 399 items.

**Table 1:** Results from Classical Test Theory & Item Response Theory Analyses of the Physical Activity (PA) Self-Efficacy Scale (n = 586)

Item How sure are you that you have (can)...			M (SD)	CITC	Factor	Infit	Est (SE)
229	x	...the ability to do other physical activities like running, dancing, bicycling, or jumping rope?	0.85 (0.35)	0.41	0.63	1.01	-1.50 (0.09)
227	x	...the ability to play team sports like basketball, soccer or softball?	0.82 (0.38)	0.41	0.61	1.02	-1.11 (0.09)
208		... be PA ≥ 30 minutes one day after school?	0.81 (0.39)	0.44	0.64	1.02	-1.00 (0.09)
230	x	...the ability to do other PA like running, dancing, bicycling, or jumping rope really well?	0.80 (0.40)	0.42	0.60	1.06	-0.99 (0.38)
224		...be PA (playing sports or games) ≥ 30 minutes one day on a non-school day, including the weekend?	0.79 (0.41)	0.42	0.60	1.06	-0.90 (0.09)
225	x	...be PA(playing sports or games) ≥ 30 minutes most non-school days, including weekend?	0.77 (0.42)	0.51	0.71	0.99	-0.73 (0.09)
209		... be PA ≥ 30 minutes at least 4 days a week after school?	0.76 (0.43)	0.49	0.68	0.97	-0.65 (0.08)
222	x	...ask your friends to be PA with you ≥ 30 minutes one day?	0.74 (0.44)	0.41	0.57	1.13	-0.51 (0.08)
228	x	...the ability to play team sports like basketball, soccer or softball really well?	0.71 (0.45)	0.50	0.69	1.02	-0.27 (0.08)
214	x	... be PA ≥ 30 minutes one day, even when you have homework?	0.67 (0.47)	0.45	0.60	1.07	-0.01 (0.08)
210		... be PA ≥ 30 minutes one day when your friends want to do something else?	0.67 (0.47)	0.50	0.66	1.03	0.01 (0.08)
223	x	...ask your friends to be PA with you ≥ 30 minutes at least 4 days a week?	0.64 (0.48)	0.46	0.61	1.08	0.19 (0.08)
216		... be PA ≥ 30 minutes one day, even when you have to stay inside the house?	0.64 (0.48)	0.48	0.63	1.06	0.24 (0.08)
226		...be PA(playing sports/games) ≥ 30 minutes most non-school days, including weekend, even if stressed?	0.63 (0.48)	0.51	0.67	1.05	0.31 (0.08)
212		... be PA ≥ 30 minutes one day when the weather outside is bad (for example, rainy, hot, or cold)?	0.60 (0.49)	0.53	0.69	0.99	0.41 (0.08)
211	x	... be PA ≥ 30 minutes at least 4 days a week when your friends want to do something else?	0.59 (0.49)	0.56	0.73	0.93	0.46 (0.08)
220		... be PA ≥ 30 minutes one day, even when you are tired?	0.58 (0.49)	0.57	0.73	0.99	0.55 (0.08)
218	x	... be PA ≥ 30 minutes one day, even when you have lots of other things to do?	0.56 (0.50)	0.49	0.63	1.02	0.65 (0.08)
215		... be PA ≥ 30 minutes at least 4 days a week, even when you have homework all month?	0.55 (0.50)	0.52	0.67	0.98	0.74 (0.08)
217		... be PA ≥ 30 minutes at least 4 days a week, even when you have to stay inside the house all month?	0.53 (0.50)	0.52	0.67	1.01	0.85 (0.08)
213	x	... be PA ≥ 30 minutes at least 4 days a week when weather outside is bad?	0.52 (0.50)	0.52	0.67	1.00	0.91 (0.08)
221		... be PA ≥ 30 minutes at least 4 days a week, even when you are tired?	0.48 (0.50)	0.57	0.73	0.91	1.17 (0.08)
219	x	... be PA ≥ 30 minutes at least 4 days a week, even when you have lots of other things to do?	0.48 (0.50)	0.59	0.77	0.89	1.19 (0.08)

% Variance Explained (Factor 1/Factor 2) (46.4%/8.5%) – Cronbach's alpha/Person-separation reliability (full scale 0.90/0.86; reduced scale 0.81, 0.78)

Corrected item total correlation [CITC]; item response modeling item difficulty estimate & standard error [Est (SE)]; Infit statistic (acceptable range 0.75 – 1.33)

Self efficacy item scale: not sure (0), sure (1); "x" represents item retained in reduced scale

**Table 2:** Classical Test Theory & Item Response Theory Analyses of TV Computer/Video Game, and Telephone Sedentary behavior change Self-Efficacy Scale

		How sure are you that you have (can)...	**M (SD)**	**CITC**	**Factor**	**Infit**	**Est (SE)**
**Television (n = 555)**							
q235	x	...limit watching TV to 1 hour at least one school day?	0.73 (0.45)	0.42	0.61	1.20	-1.27 (0.08)
q236	x	...limit watching TV to 1 hour per day for most school days?	0.64 (0.48)	0.60	0.82	0.95	-0.61 (0.08)
q231		...not watch TV at all for one school day?	0.63 (0.48)	0.46	0.64	1.06	-0.52 (0.08)
q237	x	...limit watching TV to 1 hour at least one non-school day, including the weekend?	0.57 (0.50)	0.55	0.75	1.11	-0.10 (0.08)
q238		...limit watching TV to 1 hour most non-school days, including weekend?	0.55 (0.50)	0.65	0.87	0.90	0.09 (0.21)
q232	x	...not watch TV at all for most school days?	0.48 (0.50)	0.57	0.77	1.03	0.57 (0.08)
q233		...not watch TV at all for one non-school day, including the weekend?	0.44 (0.50)	0.60	0.80	0.95	0.83 (0.08)
q234	x	...not watch TV at all for most non-school days, including weekend?	0.41 (0.49)	0.58	0.78	0.99	1.00 (0.08)

% Variance Explained (Factor 1/Factor 2) (62.6%/13.2%) Cronbach's alpha/Person-separation reliability (full scale 0.83/0.78; reduced scale 0.74, 0.72)							

**Computer/Video Games (n = 538)**							

q243	x	...limit playing computer or video games to 1 hour at least one school day?	0.75 (0.43)	0.54	0.75	0.98	-0.70 (0.09)
q239		...not play computer or video games at all for one school day?	0.72 (0.45)	0.51	0.71	1.13	-0.44 (0.09)
q244	x	...limit playing computer or video games to 1 hour for most school days?	0.71 (0.46)	0.59	0.80	0.99	-0.35 (0.09)
q245	x	...limit playing computer or video games to 1 hour at least 1 non-school day, inc weekend?	0.69 (0.46)	0.59	0.80	0.98	-0.18 (0.09)
q246		...limit playing computer or video games to 1 hour for most non-school days, inc weekend?	0.64 (0.48)	0.58	0.78	1.02	0.19 (0.23)
q240	x	...not play computer or video games at all for most school days?	0.63 (0.48)	0.61	0.80	0.96	0.29 (0.09)
q241		...not play computer or video games at all for one non-school day, including the weekend?	0.61 (0.49)	0.59	0.78	1.02	0.44 (0.09)
q242	x	...not play computer or video games at all for most non-school days, including weekend?	0.57 (0.50)	0.64	0.85	0.89	0.75 (0.09)

% Variance Explained (Factor 1/Factor 2) (66.3%/10.1%) Cronbach's alpha/Person-separation reliability (full scale 0.85/0.79; reduced scale 0.78, 0.73)							

**Telephone (n = 520)**							

q251	x	...limit talking on the telephone to 1 hour at least one school day?	0.76 (0.43)	0.55	0.77	1.07	-0.91 (0.09)
q252		...limit talking on the telephone to 1 hour for most school days?	0.68 (0.47)	0.60	0.81	1.02	-0.27 (0.09)
q254	x	...limit talking on the telephone to 1 hour for most non-school days, including weekend?	0.68 (0.47)	0.62	0.83	0.97	-0.23 (0.24)
q253		...limit talking on the telephone to 1 hour at least one non-school day, including weekend	0.67 (0.47)	0.61	0.82	0.98	-0.15 (0.09)
q247	x	...not talk on the telephone at all for one school day?	0.65 (0.48)	0.59	0.79	1.13	0.02 (0.09)
q248	x	...not talk on the telephone at all for most school days?	0.63 (0.48)	0.46	0.63	1.20	0.21 (0.09)
q249		...not talk on the telephone at all for one non-school day, including the weekend?	0.58 (0.49)	0.58	0.77	1.04	0.58 (0.09)
q250	x	...not talk on the telephone at all for most non-school days, including weekend?	0.56 (0.50)	0.62	0.82	1.00	0.75 (0.09)

% Variance Explained (Factor 1/Factor 2) (65.9%/10.5%)

Cronbach's alpha/Person-separation reliability (full scale 0.84/0.75; reduced scale 0.75, 0.71)

Corrected item total correlation [CITC]; item response modeling item difficulty estimate [Est (SE)

Infit statistic (acceptable range between 0.75 and 1.33)

Self efficacy item scale: not sure (0), sure (1); "x" represents item retained in reduced scale

### Physical activity and sedentary behavior assessment

As part of school based monitoring of physical activity, accelerometry data were collected on 109 children, 82 of whom also provided questionnaire data. Physical activity was monitored for 5 consecutive days using the MTI actigraph accelerometer (Manufacturing Technologies Inc. Fort Walton Beach, FL). The MTI^® ^has been shown to be a reliable and valid measure of physical activity in children and adolescents [24]. Each monitor was attached to an elastic belt at the waist above the right hip. Monitors were programmed to record physical activity in 30-second intervals. The accelerometer data were aggregated in estimates of the average number of minutes engaged in sedentary, light, and moderate-to-vigorous physical activity from 3 pm to 6 pm to capture physical activity during the main period outside of school in which children can make their own physical activity decisions. Raw accelerometer data were collected in 30 second epochs that were subsequently classified as sedentary, light, or moderate-to-vigorous as determined by the intensity counts. The threshold ranges used for classification were counts between 0–50 (sedentary), 51–1499 (light), and 1500 or greater (moderate-to-vigorous) [25]. For each participant and each day, the number of 30 second epochs for each category were summed and multiplied by two to provide estimates in counts per minutes. Time not worn was determined by 5 or more minutes of consecutive zeros. Valid days were determined as a minimum of 9.51 hours and 12.51 hours of wear time (24 hours less non-wear time) for week days and weekend days, respectively. Participant estimates were obtained by averaging the number of minutes in sedentary, light, and moderate-to-vigorous activities across valid days. The mean counts per minute, which provides an indication of the total volume of physical activity in which the participant engaged [26], was also averaged across valid days for each participant and used in all subsequent analyses. Only participants with three or more valid days were included.

### Statistics

To ensure that the results were not skewed by missing data and to utilize as much information as possible a priori inclusion criterion of responses for 70% of the items within the instrument under study was applied in the analyses. We then adopted a conservative approach and imputed the item mean value [27] for participants who were missing <= 30% of the items using SPSS 15.0 for Windows [28]. The imputed values were then used in the ensuing CTT analyses. Because one of the benefits of IRM is the inclusion of participants with incomplete data, no imputation was performed for the item response modeling analyses. Frequencies and percentages were used to describe the demographic characteristics of the sample. Chi-square tests of independence and t tests for independent samples were used to examine missing data status for examination of differences between those (1) with and without some PA and inactivity SE and (2) with and without valid accelerometer data.

The evaluation of the self-efficacy instruments involved a multi-step process. Initially, traditional CTT item analysis methods were performed to examine item properties such as item difficulty (item mean and standard deviation), discrimination (corrected item-total correlation; CITC) and scale reliability (Cronbach's alpha). For the self-efficacy scales, item difficulty may be thought of in terms of the endorsement (probability associated with selecting "yes") of the item. Exploratory factor analyses (EFA) with principal axis factoring extraction were performed to assess the dimensionality of the scale and to demonstrate 'sufficient unidimensionality', i.e. the scale exhibited one primary dimension. The EFAs were performed on tetrachoric correlations because of the dichotomous nature of the data. EFAs yielded factor loadings for each of the items as well as the percent variance explained by each factor.

IRM using the Rasch model for dichotomous data was then performed [29] using ConQuest [30]. For Rasch models the ability of each item to discriminate between individuals with different trait levels on the construct of interest is assumed to be equivalent among all items [31]. The IRM procedure used all available data for participants' who provided data for at least 70% of the items and the IRM likelihood estimation and expectation-maximization algorithms were used to obtain item and person parameter estimates for all participants. This estimation procedure was used for missing data as it provides greater validity than simpler procedures such as case-wise deletion and simple imputation. However, the process relies on the assumption that data are missing at random [32].

The model utilized for physical activity self-efficacy was unidimensional whereas the model used for sedentary activity self-efficacy was a between items multidimensional with television, computer/video games, and telephone self-efficacy subscales. The between-items multidimensional model indicated that each item loaded only on one subscale. The IRM modeling process yielded the following information: item parameter estimates (item difficulty); item infit statistics; person parameter estimates (self-efficacy latent trait); the Wright map; and person-separation reliability indices. The item parameter estimate provides an indication of how hard a particular item was to achieve, for example, not watching TV at all for most non-school days, including weekends, yielded a much higher parameter estimate (1.00) than limiting TV to 1 hour per day on most school days (-0.61). The infit statistics are the extent to which the data are in agreement with the values that would have been expected from the model with ranges between zero and infinity. Values closer to 1.0 indicate more agreement between the observed and expected values. Values greater than 1.0 indicate more variation while values less than 1.0 indicate less variation. Ranges of 0.75 to 1.33 are indicative of good fit for self-reported data [20]. The Wright map provides a visual representation of the distribution of individuals on the underlying (latent) self-efficacy variable (X's on the left side of the Wright map) and the distribution of the individual items (represented on the right side by item number) on the same axis. Essentially, the left side of the Wright map is like a histogram of the person self-efficacy scores that has been rotated 90 degrees to the left. The item and person estimates are based on a standard normal distribution. Ideally, we would like to see both the person and item estimates range between -3.0 to 3.0 logits, as we are interested in developing a scale that could be used in intervention studies. The person-separation reliability index is analogous to Cronbach's alpha [33].

**Figure 1:**
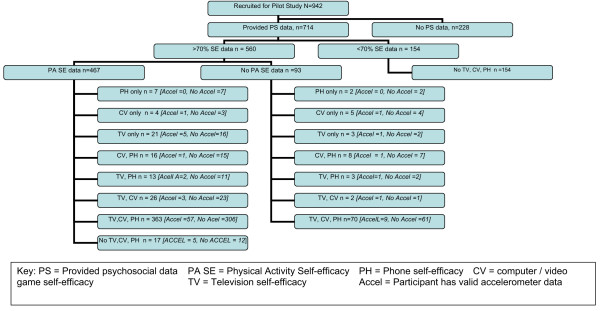
**Flow chart of participant recruitment and availability of complete and incomplete questionnaire and accelerometer data**.

Because the Wright map matches the item difficulty to the distribution of respondents on the latent trait, the Wright map identifies gaps along the self-efficacy latent continuum that were not targeted by items in the scale(s). Additionally, the Wright map identifies ranges along the continuum where the content coverage is overlapping (e.g. similar item difficulty values) [34]. To minimize participant response burden, item reduction was performed. As with CTT item analysis for criterion-referenced tests, item sensitivity (ability to discriminate) and difficulty were considered. All items are assumed to discriminate equally in the Rasch model. Because all items exhibited acceptable fit, the first step in reducing the number of items in the scale was to statistically identify items with having overlapping levels of difficulty via the Wright map. Among items with overlapping levels of difficulty the item with the highest level of difficulty was selected for inclusion in the reduced scales (Figure 1). Subsequently, the excluded item content was discussed by the research group to ensure that the excluded set of items did not exhibit a common thread (e.g., not watch TV at all) and that the validity was not threatened. The IRM was repeated on the reduced sets of items and the reliability indices expected with a shorter test were calculated using the Spearman-Brown prophecy formula. The IRM reliability as a function of self-efficacy was plotted. Although the reliability function for sedentary self-efficacy is from a between-items multidimensional model, the multidimensional graphical representation is beyond the scope of this paper. Therefore, the sedentary reliability function was viewed in a unidimensional context with separate reliability functions for each sedentary behaviour.

The complete and reduced sets of items were compared by performing paired t-tests and examining the intra-class correlation between self-efficacy estimates. Due to the influence of sample-size on the level of significance, standardized effect sizes (SEF) of the difference between item sets were also calculated. The SEF is the difference per unit of the standard deviation. Values of 0.20, 0.50, and 0.80 represent small, medium and large differences, respectively [35]. Finally correlations between each of the original self-efficacy scales and the accelerometer variables were calculated using the raw score for each self-efficacy scale. This process was then repeated using the IRM reduced scales.

## Results

Participant characteristics are shown in Table 3. Although 942 participants were initially recruited for the pilot study, only 714 participants provided useable psychosocial data (see Figure 1) and 228 participants were initially excluded because they provided no data or provided incomplete records based on information such as date of entry and ID number. Among the 714 participants, 154 participants were further excluded from the analyses because they did not complete at least 70% of the items within at least one of the questionnaires under study. Participants were categorized as missing all data if they did not complete at least 70% of the items on at least one of the physical activity and inactivity self efficacy questionnaires. Results from chi-square tests of association between missing data status (< 70% of items completed) and demographic characteristics yielded a significant [*X*^2^(3) = 14.17, p = .003] association between missing data status and race/ethnicity. However, the contingency coefficient (C = 0.13) showed that the association was small. Hispanic participants were more likely than White [OR = 1.9 (1.2, 2.8)] and Black participants [OR = 1.4 (1.0, 1.9)] to have all missing data. Because the probability of missing is more likely to depend on race/ethnicity and less likely to depend on PA or inactivity self-efficacy, the data were considered to be missing at random.

**Table 3:** Participant Characteristics

Characteristic	Missing data status group	
	
	Missing all SE and accelerometer data	Some or complete SE data with or without accelerometer data
Total^a ^*n (%)*	382 (40.6)	560 (59.4)
Gender *n (%)*		
Male	193 (50.5)	276 (49.3)
Female	187 (49.0)	276 (49.3)
Missing^b^	2 (0.5)	8 (1.4)
Race/Ethnicity* *n (%)*		
White	37 (9.7)	87 (15.5)
Black	88 (23.0)	155 (27.7)
Hispanic	214 (56.0)	270 (48.2)
Other	40 (10.5)	38 (6.8)
Missing^b^	3 (0.8)	10 (1.8)
Highest education for head of household *n (%)*		
HS graduate or less	208 (54.5)	261 (46.6)
Some college or specialized training	95 (24.9)	159 (28.4)
College graduate	51 (13.4)	68 (12.1)
Missing^b^	28 (7.3)	72 (12.9)
Age (in years) *n: M (SD)*	380: 11.3 (0.6)	552: 11.3 (0.6)
BMI%tile *n: M (SD)*	377: 73.2 (27.9)	552: 70.5 (28.1)

^a ^Total percents are displayed as row percents; remaining percents are displayed as column percents

^b ^Missing not included in chi-square tests of association

* Significant [*X*^2^(3) = 14.17, p = .003] association between missing data status and race/ethnicity; however, the contingency coefficient (C = 0.13) showed that the association was small. Hispanic participants were more like than White participants [OR = 1.9 (1.2, 2.8)] and more likely than Black participants [OR = 1.4 (1.0, 1.9)] to have all missing data

Of the 109 participants who provided accelerometer data, only 88 participants were included in the final validation analyses involving the correlations between behavior and self-efficacy; the remaining 27 participants were excluded because they did not provide any PA or inactivity self-efficacy data. There were no significant differences between the participants with valid accelerometer and some PA and inactivity self-efficacy data and those without (n = 860).

The results of CTT and IRM applied to the physical activity self-efficacy data are shown in Table 1. The item mean indicates the difficulty of the item and the results have been presented to list the items in descending order of difficulty with "the ability to do other team sports like running, dancing, bicycling or jumping rope" being the most difficult item. The corrected item total correlations (CITC) indicate the extent to which the item can discriminate between participants with low and high physical activity self-efficacy. All of the CITC scores were above 0.41 (scores that are greater than 0.30 [36] are considered to be excellent). Factor analysis indicated that a one factor solution explained 46.4% of the variance in the items with the two factor solution explaining only 8.5% more of the variance. The factor analysis therefore showed that all of the items loaded onto one factor and that the instrument was assessing a cohesive construct. Therefore the assumption of sufficient unidimensionality was satisfied. The individual factor loadings for each item when the dominant one factor solution was used ranged from 0.57 to 0.73 and the alpha for this scale was 0.90 suggesting that the items were assessing the same construct.

Also included in Table 1 is the infit statistic based on the statistical modeling of the obtained data in relation to the statistical expected values. Inspection of the infit values indicated that all of the physical activity self-efficacy items infit indices were between 0.89 and 1.13, well within the range of acceptable fit (0.75 – 1.33) [20,29], thus indicating the observed parameter estimates are close to what was expected and the physical activity self efficacy (the latent variable) fit the item.

**Figure 2:**
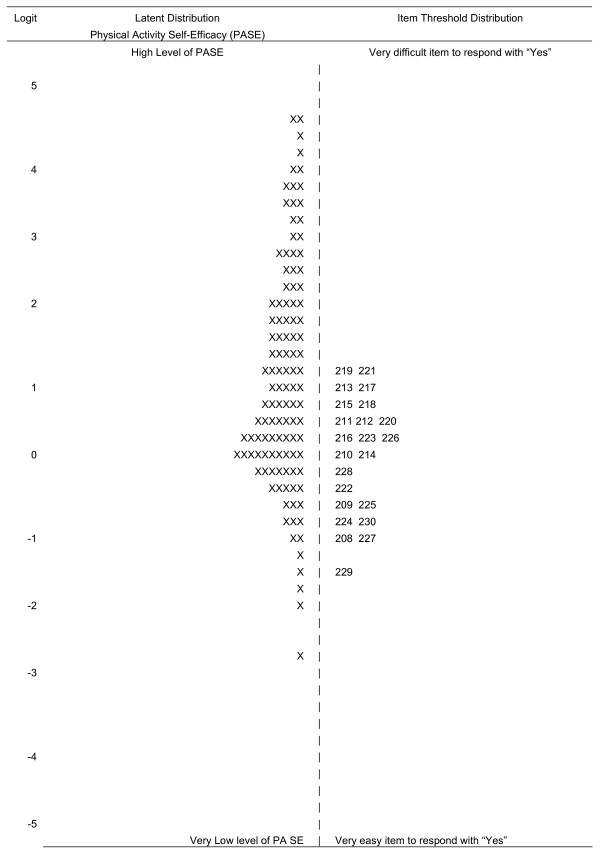
**Wright Map of Physical Activity Self-Efficacy Latent Distribution and Item Difficulty Estimates, with each "X" representing 5.0 cases**.

The left side of the Wright map (Figure 2) displays the distribution of participants (with each X representing 10 participants) while the right side represents the distribution of items, both along the latent self-efficacy variable. The number of items in the questionnaire represents the position of the item along the difficulty dimension. The scale is presented in logits which are comparable to the log of the odds ratio of the recorded responses predicting the expected response with 0 being the center of the difficulty of items. The distribution of participants on self-efficacy was skewed towards higher values. This is evident as there are a large number of participant scores (represented as X's) at values of zero and above. There are very few scores below zero. The right side of the figure indicates the difficulty of each of the items retained in the scale. The distribution of items indicated no item difficulty estimates covered the scores that were extremely easy (<-2.0 logits) or extremely difficult (>2,0 logits). Furthermore, the Wright map showed that although participants exhibited higher self efficacy scores, as evident by the X's located at approximately between 1.5 and 4.5 logits, there were no item difficulty estimates above 1.5 logits.

**Figure 3:**
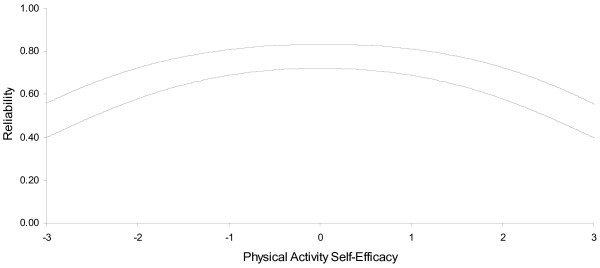
**Reliability as a function of physical activity self efficacy; full set of items (solid line) and reduced set of items (dashed line)**.

The distribution of items in Figure 2 also indicates overlapping items at multiple points along the underlying variable. This suggests that the number of items could be reduced, yet still cover the segment of the distribution already covered. The 12 items indicated with an X in the second column of Table 1 were retained from the original 22 in the final abbreviated scale. This reduced set of items was generated by selecting the one item with a higher item difficulty in each group of two or more overlapping items in a row in Figure 2. The reliability (shown in Figure 3) is plotted as a function of self-efficacy. The person reliability for the full PASE scale approximated 0.8 at its apex (Figure 1) but closer to 0.6 in its tails. Person reliability for the reduced PASE scale approached 0.7 at its apex, and 0.4 in the tails. Composite measures of physical activity self-efficacy for the full scale were 0.90 and 0.86 for Cronbach's alpha and the IRM person-separation reliability, respectively. As expected, the reliability decreased for the reduced scales to 0.81 and 0.78 for Cronbach's alpha and the IRM person-separation reliability, respectively. These reduced values were as expected based on the Spearman-Brown prophecy formula (values not shown), thus indicating the reduction in reliability was due to a decrease in the number of items [37].

The item characteristics for each of the three sedentary behavior change scales are shown in Table 2. For television viewing the mean CTT-derived item mean difficulties ranged from 0.73 for "limit watching TV to 1 hour on at least one school day" to 0.41 for the item "not watch TV at all for most non-school days, including weekend days" and the CITC scores were all above 0.42. Factor analysis indicated that a one factor solution accounted for 62.6% of the variance while the second factor only accounted for 13.2% more of the variance. Therefore the assumption of sufficient unidimensionality was satisfied. All of the items had infit statistics between 0.89 and 1.20 that were well within the range of acceptable fit, thus indicating that the difference between the observed and expected item difficulty was reasonable. The item difficulties for the television viewing scale indicated that the 9 items could be grouped into five different levels of self-efficacy (Table 1). It is noticeable; however, that there was a limited spread of difficulty scores for the items with no very difficult or very easy self-efficacy items. As such, the scale was not able to capture the complete spectrum of TV viewing self-efficacy (Figure 3).

**Figure 4:**
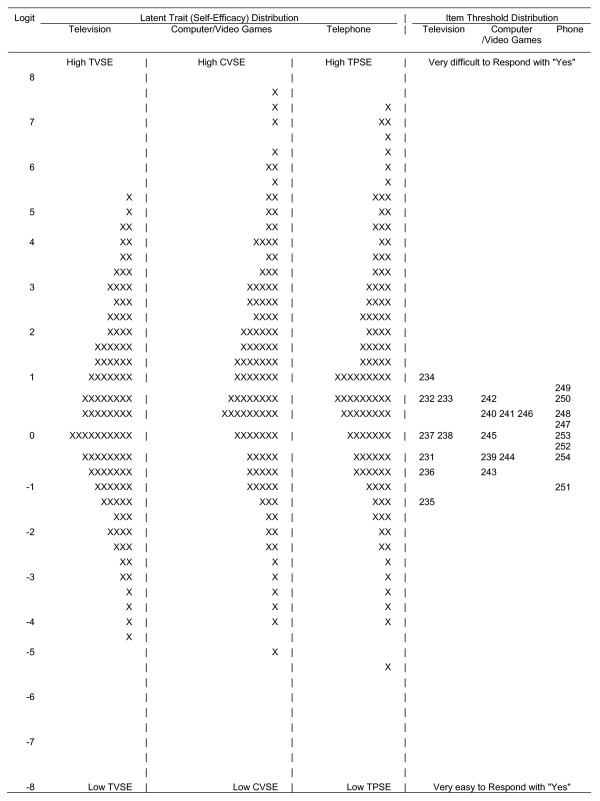
**Wright Map of Television Computer/Video Games and Telephone Self Efficacy Latent Distribution and Item Difficulty Estimates, with each "X" representing 5.1 cases**.

**Figure 5:**
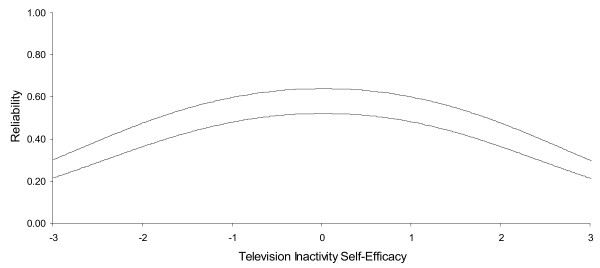
**Reliability as a function of television inactivity self efficacy; full set of items (solid line) and reduced set of items (dashed line)**.

Sufficient unidimensionality for computer/video games was established with EFA results. The one factor solution accounted for 66.3% of the variance while the second factor only accounted for 10.1%. The CITC scores were all above 0.54; the factor loadings were above 0.71; and the alpha was 0.85. The infit ratios were all in the desired range and could be reduced to five items, but like TV viewing the items did not provide a wide spread of computer/video game playing difficulty. A similar pattern was also observed for telephone self-efficacy which was a single factor, had good internal consistency (alpha = 0.84), could be reduced to five items, but did not include sufficiently difficult or easy options to assess the full range of participant self-efficacy in relation to this behavior (Table 2 and Figure 4) The person related reliability for the full television watching change self efficacy scale approached 0.6 at its apex but approximated 0.3 in its tails (Figure 5).The person related reliability for the reduced television change self efficacy scale approached 0.5 at its apex, but 0.2 in its tails. The poor reliability of the reduced set of items suggests that the reduced set of television change items are not sufficiently reliable for use and therefore more work is needed to enhance the reliability of this scale.

The person related reliability curves for the computer videogame and telephone change scales were virtually identical to those for television change self efficacy, and were not reproduced here.

Descriptive statistics for the mean IRM scores obtained for each scale for both the full and reduced set of items and associations with the accelerometer data are shown in Table 4. The association between physical activity self-efficacy and accelerometer counts per minute was the same (r = 0.18) for the boys for both the full and reduced set of items, but not statistically significant. Similar non-significant associations for the girls (r = 0.16 and 0.15) were observed. Associations were lower (r = 0.09 – 0.11) with moderate to vigorous physical activity. Television viewing behavior change self-efficacy was positively associated with light intensity physical activity (r = 0.33) for the boys and negatively associated with sedentary time (r = -0.29) when using the full set of IRM items. Weaker non-significant associations were obtained for the girls (r = -0.05 – 0.09). This pattern was also evident for computer use behavior change self-efficacy with both the full and reduced set of items. Similarly, for phone use behavior change self-efficacy the full set of items was positively associated (r = 0.17) with light intensity activity for the boys and negatively associated with sedentary time (r = -0.12) but associations were much weaker for the girls (r = -0.04 and 0.05). Re-running the analysis comparing the items for the IRM derived scale and the classical test theory (CTT) raw mean yielded similar results. (Data not in tabular form).

**Table 4:** Complete sample scale means, standard deviations, intra-class correlations (ICC), and sub-sample correlations between physical activity/sedentary behavior change self-efficacy scores and afternoon physical activity

Self-Efficacy Scale		Complete Sample		Sub-sample (Includes participants with valid accelerometer data)					
		Scale	ICC	Correlations					
					Males			Females	
	n	M (SD)		n	CPM	MV	n	CPM	MV

Physical Activity Self-Efficacy*	586			41			47		
Full Item Scale		1.07 (1.58)	0.93		0.18	0.09		0.16	0.09
Reduced Item Scale		1.15 (1.36)			0.18	0.11		0.15	0.09
					Light	Sedentary		Light	Sedentary
Sedentary behavior change Self-Efficacy	555			37			46		
Television Self-Efficacy									
Full Item Scale		0.44 (2.04)	0.96		0.33*	-0.29		0.09	-0.05
Computer/Video Games	538			37			41		
Full Item Scale		1.30 (2.26)	0.97		0.27	-0.21		0.09	-0.04
Telephone*	520			35			39		
Full Item Scale		1.23 (2.30)	0.98		0.17	-0.12		-0.04	0.05

Abbreviations: item response modeling (IRM)-derived estimates; intra-class (ICC) correlation between full and reduced estimates; average daily accelerometer counts per minute (CPM); moderate-to-vigorous (MV) minutes between 3 pm to 6 pm; sedentary (sedentary) minutes between 3 pm and 6 pm; light (light) minutes between 3 pm and 6 pm;

Notes: No significant differences between self-efficacy scores for participants with and without accelerometer data

* Significant difference between full and reduced scores at p <.0.05; However, IRM-derived differences were very small (standardized effect sizes of 0.00–0.15)

## Discussion

Item response modeling was used to assess the psychometric characteristics of new physical activity and sedentary behavior change self-efficacy scales among 6^th ^grade students, predominantly from ethnic minority groups in seven communities across the US. The reduced item scales had fewer questions than comparable existing measures [18]. Self-efficacy is a key construct of Social Cognitive Theory (SCT) which has been used to design a large number of youth physical activity [6,15,38] and sedentary behavior change interventions [39,40]. Participant burden is a key issue for ethics committees [41] and like most investigators, members of this writing team have been asked to reduce the number items that participants are asked to complete. Such requests often force investigators to make strategic decisions about what constructs to assess. Unfortunately, potentially informative or theoretically important items were not included which limits our ability to fully understand the dynamics of youth physical activity. The data presented in this paper have shown that applying item response modeling to questionnaires can reduce participant burden by identifying items with comparable levels of difficulty and eliminating redundancy, but maintaining desirable psychometric characteristics. Through this process we have developed a reliable reduced set of items for physical activity self-efficacy but unfortunately this process did not yield reliable reduced scales for television watching, computer game and telephone change self-efficacy and thus more work is needed to refine these scales.

The four scales produced in this study had excellent internal consistency and the factor analysis showed that items included in each scale were assessing the same construct. The high loadings and internal consistency of these scales compare favorably to similar self-efficacy scales such as the Saunder's [18] self-efficacy scale which had three sub-scales and alphas that ranged from 0.52 to 0.71. In earlier work, a CTT adaptation of Saunders self-efficacy scale that included both physical activity and sedentary items correlated 0.18 with accelerometer derived MVPA, 0.13 with light intensity physical activity and -0.16 with sedentary time for the boys [14]. Thus, the associations obtained here were slightly weaker for MVPA, but by using specific sedentary behavior change questions we were able to obtain better associations with light intensity physical activity and sedentary time for the boys. However, while the associations with the physical activity self-efficacy scale were comparable for both genders the sedentary behavior change self-efficacy scales were all poorly associated with light intensity physical activity and sedentary time among the girls. Therefore, these findings show that although our new questionnaires have a more cohesive, single factor structure, good internal consistency and are more closely associated with the sedentary behaviors of interest than existing scales for the boys, they are not an improvement for considering these constructs in girls. This is important because a number of interventions that have attempted to increase physical activity by increasing self-efficacy have reported limited effects on physical activity and little or no effects on self-efficacy among girls [6,42,43]. This failure could be at least partially attributable to a lack of precision in the self-efficacy measure. Thus, while using the new IRM scale might improve our ability to detect predictors of sedentary behavior change among the boys, more work is needed to develop improved scales that are more closely associated with girls' behaviors.

The Wright maps show that despite our best effort the difficulty of the items was truncated. Our new scales did not include items that were sufficiently difficult nor easy to fully assess the potential variability in youth physical activity and sedentary self-efficacy. The full physical activity self efficacy scale (with a substantially larger number of items) had good levels of reliability over most of the range of the scale. The reduced item PASE had acceptable reliability in the center of but was low in the tails. The full television change self efficacy scale (with a larger number of items) had low levels of reliability over the full range of the scale. The reduced item television change self efficacy scale had even lower reliability at all points along the distribution. The same was found for the computer videogame and telephone change self efficacy scales. Developing and testing more items for the tails of these distributions are necessary to enhance both their reliability and the content validity. Thus, while our scale can be used to provide information about physical activity self-efficacy, researchers need to be aware of this limitation.

One way to achieve a greater range in item difficulty could be to change the response options from dichotomous to a longer Likert scale, however, although this approach had some utility for adults, in earlier childhood research we found that Likert style responses did not yield additional information among children [22]. Thus, an even bolder approach to questionnaire design that includes very easy and very difficult self-efficacy items may be needed. Such items might include a participant's perceived ability to engage in an hour per day of physical activity even when all other factors such as the environment (heat, rain or cold, etc.), school pressures (homework), other commitments (friends, non-active clubs, family activities, etc.), and general time related issues make being active very difficult. More work is needed, particularly for girls.

Strong associations (all ≥ 0.70) were obtained between all of the sedentary behavior change self-efficacy scores. This suggests that participants who felt confident in their ability to limit TV viewing also felt able to reduce their video game playing and telephone use. The strong associations between these three measures may suggest that sedentary behavior self-efficacy is a more general trait and therefore strategies to change all three behaviors may be more effective than those that just target an individual behavior. As sedentary alternatives to TV are becoming extremely popular, it may be necessary to target all three to meaningfully reduce sedentary behavior. Since previous interventions have shown that reducing TV viewing is an effective method of improving youth body composition [39,44], new interventions that focus on enhancing self-efficacy for a broader range of sedentary behaviors appear promising.

### Strengths/limitations

This study developed and tested new item response modeled physical activity and sedentary behavior change self-efficacy scales. The utility of these scales was enhanced by validating them in a diverse sample of youth that includes a high proportion of minority adolescents from across the United States. The higher levels of missing data from the Hispanic participants limits our ability to draw conclusions about the representative nature of our data and indicates that replication of our work is warranted, particularly with Hispanic youth.

The lack of accelerometer data for a significant proportion of our participants also limited our ability to compare the validity of these new scales to the validity of published scales. However, as there were no significant differences between participants who provided and did not provide accelerometer data it is reasonable to assume that the participants who provided accelerometer data were broadly representative of all of the participants included in this study. The low validity correlations among girls indicate more formative and developmental research is needed in this group.

## Conclusion

Item response modeling produced physical activity and sedentary behavior change self-efficacy scales which have fewer items and superior internal consistency than existing classical test theory derived alternatives. The items not covering the full length of variation among participants indicates that more work is needed. The new scales had reasonable validity for boys, but more work is needed to develop comparably or more valid scales for girls. Utilizing these scales in interventions may provide greater insights into the extent to which self-efficacy functions as a mediator of physical activity behavior change among adolescents and the utility of designing interventions to change physical activity and sedentary behavior self-efficacy.

## Competing interests

The authors declare that they have no competing interests.

## Authors' contributions

The paper was devised by RJ and TB. RJ wrote the first draft of the paper. KW performed all analyses. CB performed the initial development of the survey items assisted by and then refined by RJ, TB, JB and DT. All authors assisted with the overview of data collection, the presentation of the data and commented on drafts of the manuscript.
